# Systemic administration of Resolvin D1 reduces cancer‐induced bone pain in mice: Lack of sex dependency in pain development and analgesia

**DOI:** 10.1002/cam4.70077

**Published:** 2024-08-05

**Authors:** Alyssa Flippen, Iryna A. Khasabova, Donald A. Simone, Sergey G. Khasabov

**Affiliations:** ^1^ Department of Diagnostic and Biological Sciences, School of Dentistry University of Minnesota Minneapolis Minnesota USA

**Keywords:** antinociception, bone cancer pain, mice, Resolvin D1, sex difference

## Abstract

**Aims:**

Bone cancer produces severe pain that is treated with opioids, but serious side effects limit opioid utilization. There is therefore a need to develop effective and safe non‐opioid alternatives. The lipid mediator, Resolvin D1 (RvD1), could be a prospective candidate for cancer pain treatment. To assess RvD1 and other potential candidates, appropriate animal models that recapitulate clinical features must be used. Although several preclinical models of cancer pain have been developed, the influence of sex on the development of cancer pain and the effectiveness of RvD1 have not been studied.

**Results:**

Using a mouse model of fibrosarcoma growth in and around the calcaneus bone, we demonstrated that the mechanical hyperalgesia in the tumor‐bearing hind paw develops independently of sex, except that it developed a little sooner in female mice. A single intravenous injection of RvD1 (0.001–10 μg/kg) decreased hyperalgesia in both sexes with similar potency (ED_50_ = 0.0015 μg/kg) and efficacy. Repeated daily administration of 10 μg/kg RvD1 prolonged the analgesic effect and completely abolished hyperalgesia. This was also independent of sex.

**Conclusion:**

In this preclinical mouse model of bone cancer pain, the development of pain and the analgesic effectiveness of RvD1 are not influenced by sex.

## 1 INTRODUCTION

Pain from cancer is a severe clinical symptom that greatly affects the quality of life. Two‐thirds of cancer patients experience grievous pain and 40% report “breakthrough pain.”^1^, ^2^, ^3^, ^4^ Bone is a primary site for sarcomas^5^ and a preferential site for the metastasis of primary breast, prostate, and lung cancer.^6^, ^7^ More than 80% of the patients suffer from excruciating cancer‐induced bone pain (CIBP),^4^, ^8^ indicating that primary and metastatic bone cancers are some of the most painful types of cancer.^9^, ^10^, ^11^ Ongoing pain and hyperalgesia around bone destruction are core clinical diagnostic criteria for CIBP.^12^

The existence of sex differences in clinical pain manifestation and sex dependency in preclinical experimental pain, as well as the origins of such differences, are under intensive investigation.^13^, ^14^, ^15^, ^16^ It is well known that men and women have different sensitivity to pain, and there is a general acceptance based on clinical studies that women overall are predisposed to higher pain intensity and chronification.^17^, ^18^ However, sex differences depend on the type of painful clinical pathology.^19^ Bone cancer is more prevalent in male than in female patients.^20^ However, sex and the intensity of pain do not affect the overall survival of patients with bone cancer.^21^

Preclinical models that recapitulate basic features of CIBP have been valuable in determining mechanisms that contribute to CIBP. Studies in rodents have shown that inflammation, neuropathy, delivery of tumor‐associated pain mediators, and exosomes released from cancer cells all contribute to CIBP.^22^, ^23^, ^24^, ^25^, ^26^, ^27^, ^28^ However, most studies of CIBP in rodents were performed with males,^24^, ^27^, ^28^, ^29^, ^30^, ^31^, ^32^, ^33^, ^34^ while a few used only females.^35^, ^36^ Furthermore, very few studies compared evoked and non‐evoked pain behaviors in male and female rodents with CIBP, for example, Segelcke et al. and Falk et al.^37^, ^38^

Opioids (e.g., morphine) remain a first‐line pharmacotherapy for the treatment of severe CIBP^2^, ^39^ despite their intolerable and often life‐threatening side effects.^40^ In addition to the well‐known side effects of opioids, preclinical and clinical studies showed that opioids can increase cancer recurrence, metastases, and osteolysis during bone cancer.^41^, ^42^, ^43^, ^44^, ^45^, ^46^ These effects of opioids on cancer progression, and the current opioid crisis, underscore the need for the development of new and potent non‐opioid alternative therapeutics for the treatment of CIBP.

One potential candidate is the endogenous lipid mediator Resolvin D1 (RvD1). RvD1 is a derivative of docosahexaenoic acid (DHA), a ω‐3 polyunsaturated fatty acid (ω‐3‐PUFA). RvD1, together with other types of Resolvins (RvD2, RvD3, RvD4, RvD5, and RvD6), belongs to lipid autacoids that form a family of Specialized Pro‐resolving Mediators (SPMs). Acting through pro‐resolving pathways, RvD1 resolves inflammation, promotes tissue healing, and alleviates hyperalgesia produced by tissue injury and inflammation.^47^ Antinociceptive effects of SPMs were produced by direct application to the central^48^, ^49^ and peripheral^50^, ^51^ nervous systems, indicating that the mechanisms underlying SPM‐induced antinociception could be independent of pro‐resolving pathways. Indeed, intrathecal administration of RvD1 produced potent antinociception in a mouse model of CIBP.^48^

In this study, we used our established mouse model of bone cancer pain to determine: (1) if there is a sex difference in the development of CIBP, and (2) if antinociception produced by RvD1 is influenced by sex. We show that the development of cancer‐induced mechanical hyperalgesia, and the antinociceptive potency and efficacy of RvD1, are not dependent on sex.

## 2 METHODS

### 2.1 Animals

A total of 171 adult C3H/HeNCr mice (National Cancer Institute, Frederick, MD) were used (83 males and 88 females). Mice were housed four per cage, allowed free access to food and water, and maintained on a 12‐h light/dark schedule. At the end of the experiments, mice were euthanized by inhalation of CO_2_. The Institutional Animal Care and Use Committee of the University of Minnesota approved all experiments and procedures.

### 2.2 Bone cancer cell implantation

Murine NCTC clone 2472 fibrosarcoma cells (ATCC, Manassas, VA) were grown to confluency in 75 cm^2^ flasks in NCTC 135 medium (pH 7.4) containing 10% horse serum.

For implantation a cell suspension was created with trypsin, counted using a hemocytometer, pelleted, and resuspended in phosphate‐buffered saline. Mice were pre‐anesthetized in the anesthesia induction chamber with 2.5% isoflurane with room air. During tumor implantation, 2% isoflurane was delivered through the nose cone. Tumors were generated by injection of 2 × 10^5^ cells in 10 μL into and around the calcaneus bone of the left hind paw using a 0.3 cc insulin syringe with a 29.5‐gauge needle. Ten minutes after implantation mice recovered without signs of pain and distress. One hour after implantation, mice were returned to their home cage. None of the mice showed signs of motor dysfunction after fibrosarcoma cell implantation.

### 2.3 Drugs and injections

A stock of RvD1 dissolved in ethanol was obtained from Cayman Chemical (Ann Arbor, MI, Cat. No. 10012554) and stored at −80°C. RvD1 was diluted for intravenous (i.v.) injection in sterile saline just before the experiment. Ethanol (0.1%) in saline was used as a vehicle control. In awake mice, RvD1 or vehicle was administered via the right or left lateral tail vein.

### 2.4 Measurement of mechanical hyperalgesia

Mice were placed on an elevated mesh platform and acclimated for 20 min. Mechanical sensitivity of the plantar surface of the hind paw was determined by measuring the paw withdrawal frequency (PWF) evoked by a von Frey monofilament (Stoelting Co, Wood Dale, IL) with a bending force of 3.4 mN. This was applied to the plantar surface of each hind paw 10 times for 2 s each with approximately 5 s inter‐stimulus interval. PWF was expressed as the percentage of applications that evoked a withdrawal response. Tumor‐evoked mechanical hyperalgesia was defined as PWF ≥50%. The average withdrawal frequency for the hind paw contralateral to the tumor‐bearing paw did not exceed 30%.

### 2.5 Experimental design

#### 2.5.1 The development of mechanical hyperalgesia

Baseline values for PWF in tumor‐bearing and contralateral control paws were determined for 3 consecutive days before cancer cell implantation, on the third post‐implantation day (PID 3), and every other day up to PID 15. Mean (±SEM) PWF in tumor‐bearing and control paws were compared over time between male and female mice.

#### 2.5.2 Antinociception produced by RvD1

The antihyperalgesic effect of a single i.v. injection of RvD1 was determined in tumor‐bearing mice at PID 15–19 when mice exhibit maximal and stable mechanical hyperalgesia. Infusions of RvD1 in doses of 0.001, 0.01, 0.1, 1, 3, and 10 μg/kg, i.v., or vehicle were performed in separate groups of male and female mice (6 mice/group). Each mouse received only one injection. Four mice were used in each experimental group during the day of the experiment. Two different doses of RvD1 (or the vehicle instead of RvD1) were randomly administered i.v. at intervals of 5 min, the time required for testing mechanical sensitivity. The experimenter who performed the test was blinded to the treatment. Measures of PWF in the tumor‐bearing paw were obtained before injection, every hour for 4 h after injection, and at 24 h after injection. Because a single injection could change PWF for more than 4 h, the effects of RvD1 were calculated as the area under the curve (AUC), which provides an aggregated measure of antihyperalgesia over the 4 h test period. The magnitude of the antihyperalgesic effect was inversely related to AUC (decrease in PWF). A value of AUC from each experiment was used for calculations of median effective dose (ED_50_) and dose–response relationships. Comparisons were made between male and female mice.

To test the hypothesis that repeated injections of RvD1 will prolong the antihyperalgesia, 6 consecutive i.v. injections of 10 μg/kg RvD1 or vehicle were given at 24 h intervals (6 mice/group of both sexes). PWF in the tumor‐bearing paw was determined on PID 11, before the first injection, then on PID 12 (just before 2nd injection), PID 14 (before 4th injection), PID 16 (before the 6th injection), and on PID17, which was 24 h after the last injection.

### 2.6 Statistical analyses

Data are reported as the mean ± SEM. The hyperalgesic effect of cancer growth and the analgesic effects of multiple injections of RvD1 in male and female mice were compared by Two‐way ANOVA with repeated measures. Post hoc comparisons were made using Bonferroni's t‐tests (SigmaPlot 11.2 statistical software, San Jose, CA). AUCs of single injections of RvD1 were compared between sexes. ED_50_ and dose–response relationships were calculated and compared using nonlinear regression using consecutive extra sum of squares *F*‐test using GraphPad Prism 5 software (La Jolla, CA). Also, examples of time‐dependent effects induced by single injections of RvD1 or vehicle were compared using Three‐way ANOVA. For all statistical comparisons, a value of *p* < 0.05 was considered significant.

## 3 RESULTS

### 3.1 The development of mechanical hyperalgesia

**FIGURE 1:**
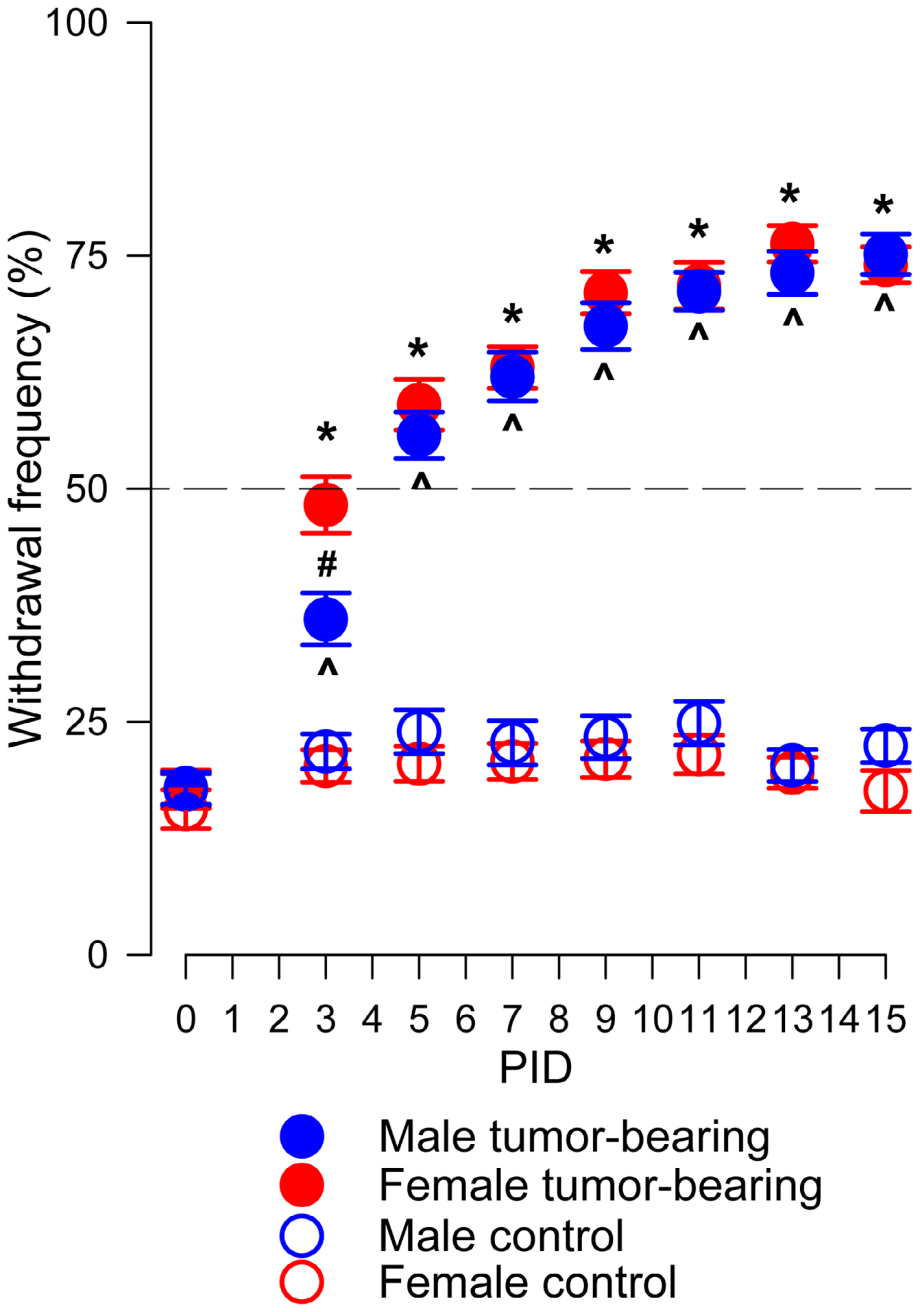
The development of bone cancer‐induced mechanical hyperalgesia in male and female mice. Fibrosarcoma cells grow in and around the calcaneus bone similarly and time‐dependently increased paw withdrawal frequency (mean ± SEM) in tumor‐bearing paws compared to baseline (0) (except early stage at PID 3). A horizontal dashed line indicates a withdrawal frequency of 50% which is considered a lower limit of mechanical hyperalgesia. Control (not injected paws) were not affected. Two‐way Repeated Measures ANOVA. Comparison with correspondent baseline: Male ˄ *p* < 0.001, female *p < 0.001, # comparison between tumor‐bearing paws of male and female, *p* < 0.001.

The development of bone cancer‐induced mechanical hyperalgesia was evaluated in 75 tumor‐bearing mice (35 male and 40 female). As shown in Figure 1, PWF gradually increased in the tumor‐bearing paw in male and female mice. Hyperalgesia reached a plateau at PID 13. Two‐way Repeated Measures ANOVA revealed a significant interaction between PWF of tumor‐bearing and contralateral paws in male and female mice over time of tumor development (PID) (*F*_(21,1199)_ = 33.799, *p* < 0.001). PWF for the tumor‐bearing paw gradually increased during tumor growth compared to baseline before implantation and starting from the PID 3 was significantly higher compared to the contralateral control paw in the mail and female mice (*p* < 0.001). There was no overall sex difference in the PWF of the tumor‐bearing paws (*p* = 0.660). However, comparison at PID 3 (*p* < 0.001) indicated a slower development of mechanical hyperalgesia at the initial stage of cancer growth in male mice. PWF of the contralateral paw did not change over time and did not differ between males and females (*p* = 0.787). We concluded that there was no significant sex difference in the development of bone cancer‐induced mechanical hyperalgesia.

### 3.2 Effects of single systemic application of Resolvin D1

**FIGURE 2:**
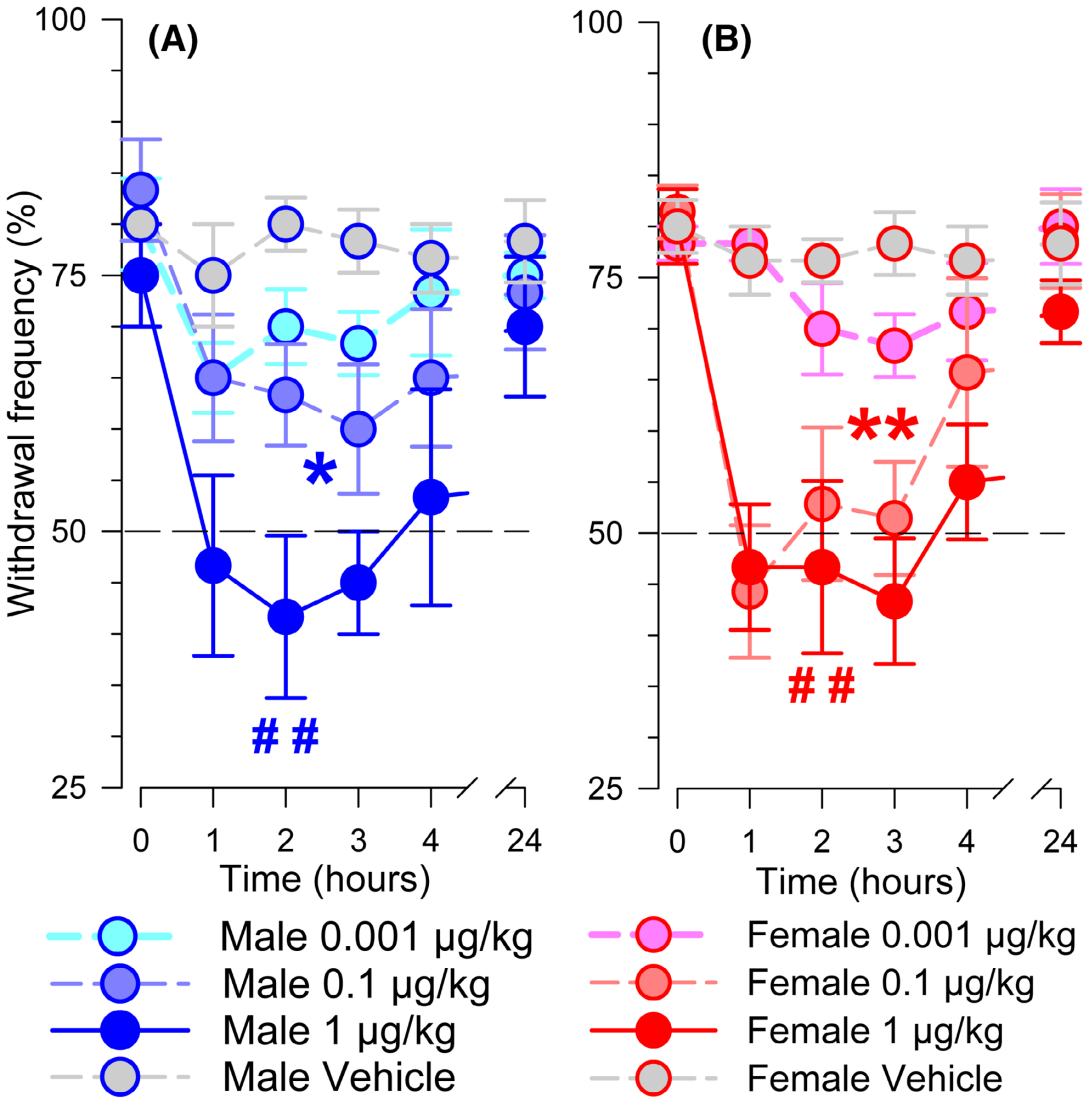
Examples of effects of i.v. injections of RvD1 or vehicle on of paw withdrawal frequency in the tumor‐bearing paw of male (A) and female (B) mice presented as mean ± SEM. The horizontal dashed line indicates a paw withdrawal frequency of 50%. Three‐way ANOVA. Comparison with vehicle: 0.1 μg/kg: *p < 0.005, **p < 0.001; 1 μg/kg: ##*s* < 0.001.

Antihyperalgesia following a single i.v. injection of RvD1 was determined in 72 tumor‐bearing mice (36 males and 36 females, 6 mice per group). Each mouse received a single i.v. injection of RvD1 at doses of 0.001, 0.01, 0.1, 1, 3, and 10 μg/kg or vehicle. In both sexes, doses of 0.1 μg/kg and higher reduced mechanical hyperalgesia which recovered in 24 h. Figure 2 shows the mean (±SEM) PWF of the tumor‐bearing paw before and after administration of 0.001, 0.1, and 1 μg/kg RvD1, or vehicle. Three Way ANOVA showed the absence of a significant interaction between sex, RvD1 dose, and the time of testing after injection (*F*_(15,293)_ = 0.697 *p* = 0.783), indicating that the antihyperalgesia following RvD1 was not influenced by sex. Administration of the vehicle did not have any effect on PWF. The 0.1 and 1 μg/kg doses of RvD1 decreased PWF in male (Figure 2A, *p* < 0.01 and *p* < 0.001) and female mice (Figure 2B, *p* < 0.001) as compared to the vehicle control. There were no differences in the antihyperalgesic effects of RvD1 between male and female mice (*p* = 0.174) (Figure 2A,B). Mechanical hyperalgesia fully recovered by 24 h after RvD1 and PWF did not differ from that obtained before injection in male (*p* = 0.680) and female (*p* = 0.908) mice.

**FIGURE 3:**
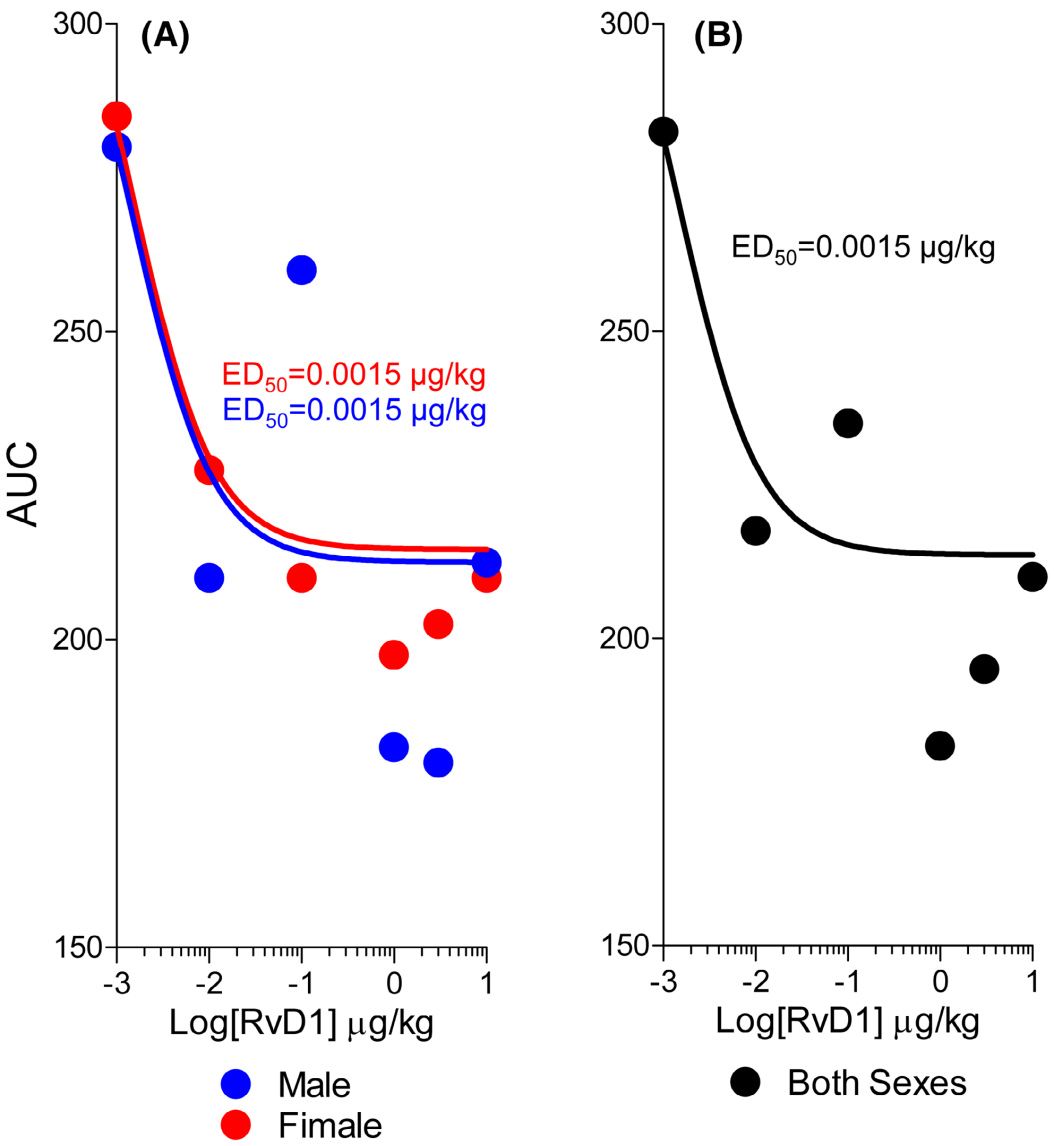
Dose‐dependent reduction of the mechanical allodynia produced by i.v. infusion of RvD1 was evaluated as the area under the curve (AUC). (A) RvD1 induced a similar dose‐dependent antihyperalgesic effect presented as Medians in the male (blue) and female (red) mice. RvD1 potency indicated ED_50_ values and did not statistically significantly differ (Extra sum‐of‐squares *F*‐test, *p* = 0.184). (B) Because of the absence of sex differences in the dose–response relationship, ED_50_, and dose response–response curves were aggregated.

Because RvD1 induced a prolonged antihyperalgesic effect, we determined an aggregate of antihyperalgesia by calculating the AUC (% × hours). As is shown in Figure 3A, the dose–response relationships of systemic administration of RvD1 in female and male mice did not differ (Extra sum‐of‐squares *F*‐test: *F* ^3,66^ = 0.01603, *p* = 0.9970). The ED_50_ for male mice was 0.0015 μg/kg (95% CI 0.0000036–0.65 μg/kg) and 0.0015 μg/kg (95% CI 0.0000023–0.96 μg/kg) for female mice. This comparison indicated that RvD1 reduced bone cancer‐induced hyperalgesia in male and female animals with similar potency and efficacy. The combined ED_50_ for mice of both sexes was 0.0015 μg/kg (95% CI 0.000021–0.11 μg/kg) (Figure 3B).

### 3.3 Effects of repeated systemic application of Resolvin D1

**FIGURE 4:**
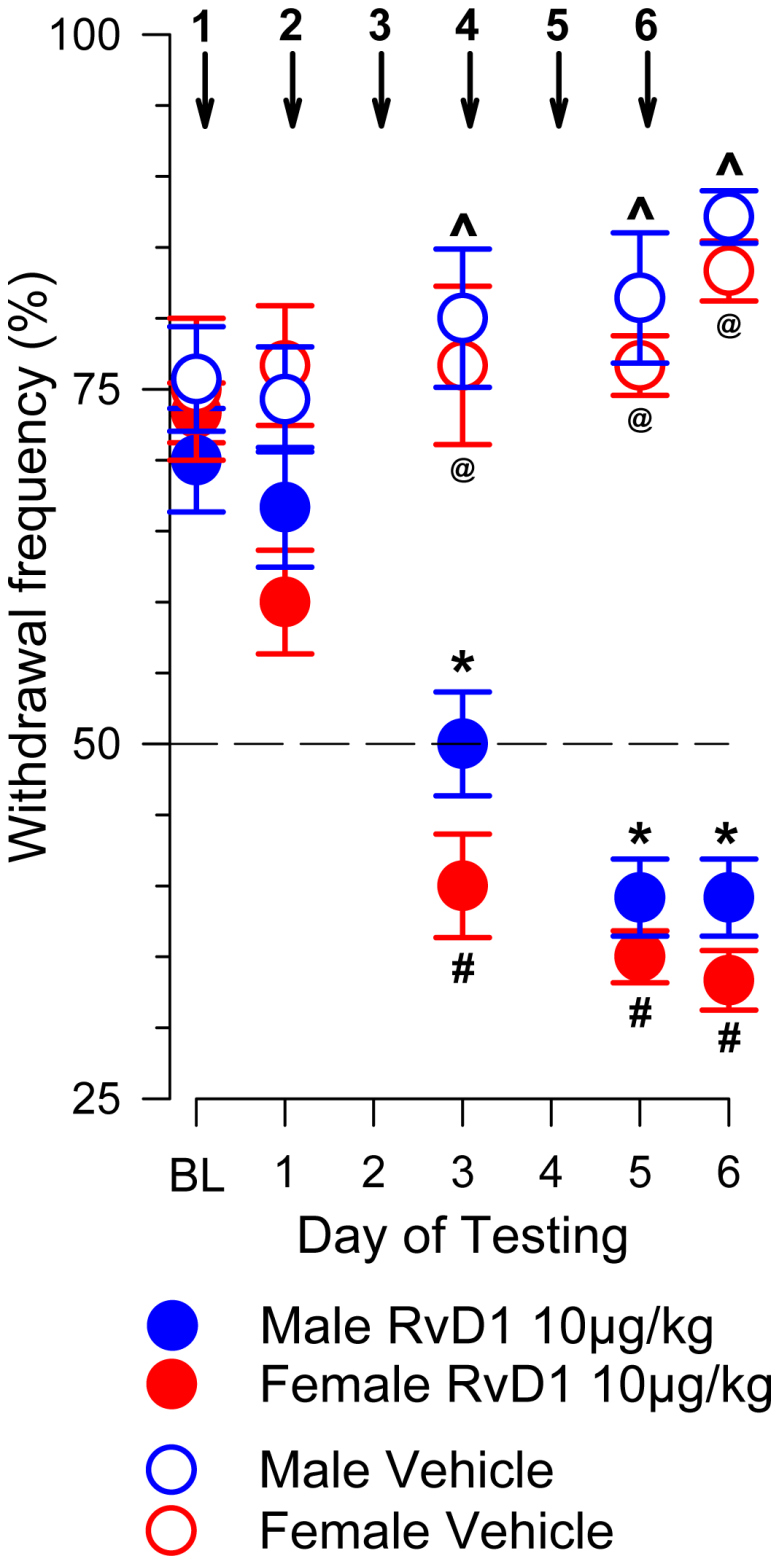
Consecutive injections of 10 μg/kg RvD1 enforced the antihyperalgesic effect. Six consecutive injections with 24 h intervals gradually prolonged and increased antinociception, eliminating hyperalgesia in tumor‐bearing male and female mice, indicated as a decrease in Mean ± SEM of PWF. Consecutive vehicle injections were without effect. The horizontal dashed line signifies a paw withdrawal frequency of 50% (hyperalgesia). Numbered vertical arrows indicate i.v. injections of RvD1 or vehicle performed every 24 h just after behavior tests. Two‐way Repeated Measures ANOVA. Changes in PWF compared to the corresponding baseline (BL) before the first injection: *male, #female, *p* < 0.001. Difference between Vehicle and RvD1 injected mice: ^male, @female, *p* < 0.001.

In 24 tumor‐bearing mice, 12 male and 12 female (6 animals per group), 6 daily i.v. injections of 10 μg/kg RvD1 or vehicle were administered at 24 h intervals. Figure 4 shows that repeated injections of 10 μg/kg, but not the vehicle, prolonged the antinociceptive effect of RvD1 and eliminated hyperalgesia in the tumor‐bearing paw of male and female mice. Two‐way Repeated Measures ANOVA indicated a significant interaction between experimental groups injected with RvD1 or vehicle and the number of injections (*F*_(12,124)_ = 12.439, *p* < 0.001). Following RvD1, but not vehicle, PWF for the tumor‐bearing paw gradually decreased in male and female mice compared to baseline measures. There was no overall sex difference in PWF injected with RvD1 (*p* = 0.884). PWF in mice given the vehicle was significantly higher compared to those that received RvD1 (*p* < 0.001). Also, there was no difference between PWF in male and female mice that received vehicle (*p* = 1.000). These data indicate that repeated administration of RvD1 produced a strong antihyperalgesic effect that was not dependent on sex.

## 4 DISCUSSION

Pain is the most common symptom in patients with advanced cancer. Clinically, bone cancer is associated with mechanical hyperalgesia.^12^, ^52^, ^53^ This association is so strong that the presence of cutaneous hyperalgesia around bone destruction is considered a core diagnostic criterion of CIBP.^12^ The existence of hyperalgesia in the experimental model we used in our experiments suggests that fibrosarcoma growth in and around the calcaneus bone is a relevant preclinical model that recapitulates clinical features of CIBP. However, to ensure that this model is valuable for preclinical studies, the influence of sex on the development of pain needed to be evaluated and compared to clinical findings. The majority of previous studies using this model were performed on male mice^24^, ^27^, ^28^, ^29^, ^30^, ^31^, ^32^, ^33^ and only a few were performed on females.^35^, ^36^ We now demonstrate that hyperalgesia to mechanical stimuli during fibrosarcoma growth in and around the calcaneus bone developed independent of sex with some minor variance. In female mice, hyperalgesia developed at an earlier stage of tumor growth. However, the magnitude of hyperalgesia did not differ between sexes. Similarly in a mouse model of CIBP produced by femoral implantation of mammary carcinoma cells sex difference in hyperalgesia development but not in final intensity in females.^38^ Interestingly, in rats with tibial implantation of mammary carcinoma, the faster development of CIBP was found in males.^37^ However, bone implantation of mammary carcinoma in rodents of both sexes cannot fully recapitulate clinical conditions. Breast cancer in men is very rare, being just 1% of all breast cancer incidents.^54^ This is not the case for fibrosarcoma development in and around the calcaneus bone.

Importantly, findings that the development of CIBP did not differ between male and female mice agree with clinical findings. There are no differences in the clinical manifestation of CIBP, including pain intensity scores, pain duration, and frequency of pain appearance between men and women.^55^, ^56^ Moreover, the lack of sex dichotomy in clinical pain is typically for the majority of painful cancers (e.g. sarcoma, lymphoma, gastrointestinal, bladder, skin, and lung cancers).^57^, ^58^, ^59^, ^60^, ^61^, ^62^ Therefore, the comparison of sex dependency in cancer pain, including CIBP, between preclinical settings and clinical manifestation indicates that the fibrosarcoma model recapitulates clinical features, making this model useful for preclinical cancer pain research.

However, in the case of orofacial soft tissue cancer, women reported more intense pain and this paralleled the greater degree of pain in female mice (as compared to male mice) implanted with a human oral cancer cell line (HSC‐3) into the tongue.^63^ This phenomenon could be attributed to the specific nature and/or localization of the tumor.

Although CIBP may develop independently of sex, the effectiveness of analgesic treatment could be affected by sex. Clinical studies suggested that women need higher doses of morphine to achieve analgesia.^64^, ^65^ Therefore, women are at a higher risk of side effects induced by opioids. This further emphasizes the need to develop new, safe, and effective treatments for cancer pain.

Potent antinociception following RvD1 has been documented in different preclinical models of pain including inflammatory pain,^66^ neuropathic pain due to nerve damage^67^ or chemotherapy,^68^, ^69^ arthritis,^70^ back pain,^71^ and postoperative pain following thoracotomy.^49^, ^72^ However, antinociception produced by RvD1 in models of CIBP, and underlying mechanisms, need further study. RvD1 as well as other SPMs may be effective for the treatment of painful cancer.^73^, ^74^ SPMs, being a non‐immune suppressant, not only decrease cancer‐induced inflammation^75^ but strongly reduce tumor growth.^74^, ^76^ Preclinical studies showed that RvD1 inhibited primary tumor growth by enhancing macrophage phagocytosis of tumor cell debris and inhibiting the release of pro‐inflammatory cytokines and chemokines from macrophages.^77^, ^78^, ^79^ Intrathecal administration of RvD1 reduced CIBP in male mice implanted with fibrosarcoma cells into the calcaneus bone.^48^ RvD2 decreased CIBP produced by sarcoma implanted into the femoral bone of males^80^ and pain induced by oral squamous cell carcinoma implanted into female mice.^78^

Our data indicate that RvD1 decreased CIBP similarly in male and female mice. There were no differences in ED_50_ and dose–response relationships between male and female mice, indicating similarities in potencies and efficacies. The aggregated ED_50_ of RvD1 for mice was 0.0015 μg/kg. The potency should be considered high compared to morphine which decreased mechanical hyperalgesia during peripheral neuropathy or visceral pain with ED_50_ of 4.4 mg/kg^81^ and 1.9 mg/kg,^82^ respectively. It was also shown that RvD1 alleviated neuropathic and inflammatory pain independent of sex, but RvD5 was effective only in male rodents.^83^, ^84^

The current study evaluated the effect of RvD1 only on stimulus‐evoked pain. However, non‐evoked spontaneous pain is also an important factor during bone cancer growth.^37^, ^38^ Moreover, different antinociceptive substances attenuate evoked and non‐evoked CIBP pain with dissimilar efficacy.^85^, ^86^, ^87^, ^88^ Therefore, the antinociceptive effect of RvD1 on non‐evoked pain as well as its sex dependency has to be determined further.

After a single i.v. injection of the highest dose of RvD1 (10 μg/kg), mechanical hyperalgesia recovered in 24 h but six daily injections of RvD1 prolonged the antihyperalgesic effect. Repeated daily treatment decreased PWF to a level below 50%, indicating complete elimination of hyperalgesia. These effects were similar in male and female groups. The reinforcement of the antihyperalgesic effect by daily administration was also shown for RvD2. A single intrathecal injection of RvD2 in male mice transiently decreased bone cancer‐induced mechanical hyperalgesia for ~3 h, whereas three consecutive daily injections decreased hyperalgesia for more than 2 weeks,^80^ possibly indicating summation in the inhibition of intracellular plasticity that is involved in neuronal sensitization. However, it should be noted that the strong antihyperalgesic effect of repeated administration of RvD1 could be attributed to the inhibitory action of resolvins on tumor growth, as demonstrated earlier.^77^, ^78^, ^79^ Our preliminary data indicate that RvD1 attenuates the fibrosarcoma growth in vivo. Therefore, it is possible that RvD1‐dependent analgesia on CIBP following multiple infusions could be dependent on two effects: (1) prolonged inhibition of nociceptive neuronal processing and (2) direct reduction of cancer growth. Both effects are aims for further investigations.

The antihyperalgesic effect of RvD1 following i.v. administration could be attributed to a decrease in peripheral and/or central sensitization. There is a consideration that mechanical hyperalgesia and allodynia are driven mainly by central sensitization.^89^, ^90^ For example, using the same model of fibrosarcoma growth in and around calcaneus bone, we showed that primary afferent nociceptors innervating skin overlying the tumor were sensitized primarily to heat,^27^, ^28^ whereas nociceptive neurons in the spinal cord exhibited robust sensitization to mechanical stimuli also.^29^ Therefore, based on these findings it could be suggested that RvD1 reduces antihyperalgesia by inhibiting central sensitization in spinal and, possibly, supraspinal neurons. In rodents, RvD1 binds to ALX/FPR2 membrane receptors,^91^, ^92^ and these receptors are expressed in the spinal cord mostly on microglia and astrocytes.^93^, ^94^ Inhibition of ALX/FPR2 receptors attenuated the antinociceptive effect of RvD1 on the neuropathic pain induced by spinal nerve ligation in rats.^67^ The neuropathic component is a part of the CIBP.^27^, ^28^ Spinal glial cells are activated during osteolytic cancer,^95^, ^96^, ^97^ increasing the release of pronociceptive cytokines/chemokines.^98^, ^99^, ^100^ In female rats with implanted mammary gland carcinoma cells into the tibial bone the inhibition of glial cells by minocycline reduced the release of cytokines/chemokines and attenuates CIBP.^97^ RvD1 inhibits the release of cytokines from the lipopolysaccharide‐activated primary microglia in vitro.^101^ Therefore, the effect on the glia could be a potential mechanism underlying ALX/FPR2 receptor‐dependent analgesia by RvD1. Mechanisms of single and multiple applications of RvD1, by which RvD1 induces analgesic effect at the level of spinal glia and neurons have to be studied in detail.

In summary, we have shown that the development of hyperalgesia following fibrosarcoma cell growth in and around the calcaneus bone is not dependent on sex. This is consistent with the development of cancer pain clinically. Therefore, this mouse model is useful for preclinical studies of CIBP. Intravenous administration of RvD1 produced a potent reduction of cancer‐induced hyperalgesia in male and female mice and should be considered a candidate for treating CIBP.

## AUTHOR CONTRIBUTIONS

**Alyssa Flippen:** Formal analysis (equal); investigation (equal); methodology (supporting); validation (supporting); visualization (equal). **Iryna A. Khasabova:** Data curation (equal); formal analysis (equal); methodology (equal); validation (equal); writing – review and editing (equal). **Donald A. Simone:** Conceptualization (equal); funding acquisition (equal); methodology (equal); supervision (supporting); writing – review and editing (equal). **Sergey G. Khasabov:** Conceptualization (lead); data curation (equal); formal analysis (lead); funding acquisition (equal); methodology (lead); project administration (lead); resources (lead); validation (equal); writing – original draft (lead); writing – review and editing (equal).

## FUNDING INFORMATION

This work was supported by NIH grants CA263777 (SGK) and CA241627 (DAS).

## CONFLICT OF INTEREST STATEMENT

The authors declare that they do not have competing financial interests or personal relationships that could affect experimental results or conclusions reported in this manuscript.

## Data Availability

The data that support the findings of this study are available from the corresponding author upon reasonable request.
